# Massive Hemorrhage in PCV Is Associated With Lower‐Than‐Normal VEGF Level and Dramatically Elevated Inflammatory Cytokine Levels in Aqueous Mingxuan

**DOI:** 10.1155/joph/9272271

**Published:** 2025-12-29

**Authors:** Mingxuan Zhang, Yanling Wang, Lina Feng, Pei Zhang, Yingyu Li, Huijin Chen

**Affiliations:** ^1^ Peking University Third Hospital, Beijing, China, puh3.net.cn; ^2^ Department of Ophthalmology, Peking University Third Hospital, Beijing, China, puh3.net.cn; ^3^ Beijing Key Laboratory of Restoration of Damaged Ocular Nerve, Beijing, China

**Keywords:** aqueous humor, cytokines, massive hemorrhage, polypoidal choroidal vasculopathy

## Abstract

**Purpose:**

To compare the aqueous cytokine profile in polypoidal choroidal vasculopathy (PCV) with or without massive hemorrhage and typical neovascular age‐related macular degeneration (nAMD).

**Methods:**

The present comparative study included 81 treatment‐naïve eyes from 75 patients (PCV with massive hemorrhage 20 eyes, PCV without massive hemorrhage 19 eyes, typical nAMD 19 eyes, and cataract control 23 eyes). 10 cytokines (VEGF, IL‐6, IL‐8, MCP‐1, ICAM‐1, VCAM‐1, IP‐10, G‐CSF, and IFN‐γ, IL‐10) in the aqueous humor were measured by cytometric bead array.

**Results:**

VEGF levels in PCV with massive hemorrhage (median 5.20 pg/mL) were significantly lower than that in PCV without massive hemorrhage (median 34.76 pg/mL, *p* = 0.003) and typical nAMD (median 43.88 pg/mL, *p* < 0.001). They were even lower than that in normal cataract controls (median 22.02 pg/mL, *p* = 0.037). Multiple inflammatory cytokines were dramatically elevated in PCV with massive hemorrhage. IL‐6, IL‐8, MCP‐1, ICAM‐1, and IP‐10 levels were significantly higher in PCV with massive hemorrhage than that in the other three groups. VCAM‐1 levels were significantly higher in PCV with massive hemorrhage than that in typical nAMD and in control. G‐CSF and IFN‐γ levels were significantly higher in PCV without massive hemorrhage than that in control. IL‐10 levels were significantly higher in PCV with massive hemorrhage than that in typical nAMD.

**Conclusions:**

This pilot study showed that PCV with massive hemorrhage had a lower‐than‐normal VEGF level in aqueous humor and inflammation may be actively involved in the pathogenesis of massive hemorrhage in PCV.

## 1. Introduction

Polypoidal choroidal vasculopathy (PCV) is a serosanguinous disease characterized by orange‐red subretinal elevations on fundus examination, polypoidal lesions on indocyanine green angiography (ICGA), and sharp peak pigment epithelium detachment (PED) on optical coherence tomography (OCT), which frequently lead to exudative and/or hemorrhagic complications in the subretinal pigment epithelial space [1–4]. Whether PCV is a subtype of neovascular age‐related macular degeneration (nAMD) or a fundamentally different form of disease remains controversial [5, 6]. Although the natural course of PCV is reported to be more favorable than nAMD in some literature, other studies have suggested that PCV has a much higher incidence for massive subretinal hemorrhage and breakthrough vitreous hemorrhage than nAMD, which usually cause a very poor visual prognosis [7–12].

Vascular endothelial growth factor (VEGF) is one of the most crucial factors in the pathogenesis of choroidal neovascularization (CNV) in nAMD through its angiogenic activities [13, 14]. Numerous studies have showed the positive effects of anti‐VEGF therapy in the treatment for nAMD [15–18]. However, it has been found that PCV responds less successfully to the anti‐VEGF therapy [19–21]. Moreover, vascular endothelial cells of the surgically excised specimen of PCV appeared to be lack of VEGF immunopositivity [22], and levels of aqueous VEGF in eyes with PCV were found to be significantly lower than the eyes with nAMD in one study [23]. These evidences suggest that the development of PCV maybe less likely to be dependent on VEGF‐related pathways.

Elevated inflammatory cytokines have also been reported in PCV [24–27]. Vitreous samples obtained from eyes with PCV undergoing vitrectomy for vitreous hemorrhage have been found to have 10‐fold higher levels of IL‐1β expression than control eyes and were also higher than eyes with CNV‐AMD [27]. Furthermore, the surgically excised specimen of PCV showed intense infiltration of plasma cells and lymphocytes [28]. All these implies that inflammation may play an important role in the pathogenesis of PCV.

Despite all these important findings of previous studies, to the best of our knowledge, there has been no report focusing on VEGF and inflammatory cytokine levels in eyes with massive hemorrhage secondary to PCV, which is the most unfavorable phenotype of PCV. The present study aims to investigate cytokine levels in PCV with massive hemorrhage and compare them with that in PCV without massive hemorrhage, typical nAMD, and cataract controls. This study may shed some light on the pathogenesis of the severely hemorrhagic type of PCV.

## 2. Materials and Methods

### 2.1. Patient

Twenty PCV eyes (19 patients) with massive hemorrhage, 19 PCV eyes (19 patients) without massive hemorrhage, 19 typical nAMD eyes (16 patients), and 23 cataract control eyes (21 patients) without other retinal disorders were included from the Department of Ophthalmology, Peking University Third Hospital, between August 2021 and January 2024. All eyes were treatment naive. In the group of PCV without massive hemorrhage, 17/19 eyes were exudative type without hemorrhage and 2/19 had hemorrhage smaller than 4 disc diameter (DD). In the group of PCV with massive hemorrhage, all eyes had hemorrhage extending beyond the temporal arcades and massive hemorrhage was defined as extensive subretinal hemorrhage greater than 10 DD and hemorrhagic PED larger than 4 DD ±vitreous hemorrhage. Only 1 out of 20 eyes in PCV with massive hemorrhage suffered from peripheral PCV; the other 19 eyes in PCV with massive hemorrhage and 19 eyes in PCV without massive hemorrhage all had central PCV lesions. This study was reviewed and approved by the Hospital Ethics Committee and adhered to the tenets of the Declaration of Helsinki. Written informed consent was obtained from all subjects. There were no patient and public involvement in this research.

At baseline, all patients underwent comprehensive ophthalmic examination including best‐corrected visual acuity (BCVA), noncontact tonometry, slit lamp‐assisted biomicroscopy of the anterior segment of the eye, and indirect ophthalmoscopy of the posterior segment of the eye. The differential diagnosis of PCV and nAMD was based mainly on ICGA, together with other multimodal imaging including color fundus photography, fundus fluorescein angiography (FFA), spectral domain or swept source OCT, and OCT angiography (OCTA). In the presence of a dense vitreous hemorrhage obscuring the media, PCV with massive hemorrhage was diagnosed on the basis of preoperative B ultrasonographic findings showing extensive subretinal hemorrhage, as well as intraoperative observation of both extensive subretinal hemorrhage and large hemorrhagic PED, with or without orange‐red subretinal elevations, which were all directly visualized after the neural retina was flipped in all cases with massive hemorrhage.

### 2.2. Acquisition of the Aqueous Humor Samples

Undiluted aqueous humor samples (50–100 μL) were collected through anterior chamber paracentesis at the beginning of vitrectomy/cataract surgery or just before the intravitreal injection. All procedures and sample collections were performed using a standard sterilization procedure that included the use of topical povidone–iodine and levofloxacin drops. Samples were collected in sterilized plastic tubes and centrifuged at 3000 rpm for 10 min at 4°C to remove cells and debris and then immediately transferred to store at −80°C until use.

### 2.3. Measurement of Cytokines

Cytokine concentrations were determined with the flow cytometric bead array (CBA) method using the following multiplex kits (BD Biosciences, 10975 Torreyana Rd. San Diego, CA 92121): BD™ CBA Human VEGF Flex Set (Cat. No.: 558336), BD™ CBA Human IL‐6 Flex Set (Cat. No.: 558276), BD™ CBA Human IL‐8 Flex Set (Cat. No.: 558277), BD™ CBA Human MCP‐1 Flex Set (Cat. No.: 558287), BD™ CBA Human Soluble ICAM‐1 Flex Set (Cat. No.: 560427), BD™ CBA Human Soluble VCAM‐1 Flex Set (Cat. No.: 560427), BD™ CBA Human IP‐10 Flex Set (Cat. No.: 558280), BD™ CBA Human G‐CSF Flex Set (Cat. No.: 558327), BD™ CBA Human IFN‐γ Flex Set (Cat. No.: 558269), BD™ CBA Human IL‐10 Flex Set (Cat. No.: 558274), and BD™ CBA Human Soluble Protein Master Buffer Kit (Cat. No. 558265). The CBA method of capturing a soluble analyte or a group of analytes with known size and fluorescent beads enables the detection of analytes by flow cytometry. Each capture bead has been conjugated to a specified antibody. The detection reagent is a mixture of phycoerythrin (PE)‐coupled antibodies that provide a fluorescent signal proportional to the amount of bound analyte. When capture beads and detector reagent are incubated with an unknown sample containing recognized analytes, sandwich complexes (capture bead + analyte + detection reagent) are formed that can be measured using flow cytometry (BD FACS Calibur, BD Bioscience, San Jose, CA) to identify particles with the fluorescence characteristics of the bead and detector. Totally, 10 angiogenic and inflammatory cytokines (VEGF, IL‐6, IL‐8, MCP‐1, ICAM‐1, VCAM‐1, IP‐10, G‐CSF, IFN‐γ, IL‐10) in the aqueous humor were analyzed.

### 2.4. Statistical Analysis

All data were analyzed by using the Statistical Package for the Social Sciences statistical software for Windows, version 27.0 (SPSS Inc.). Differences of cytokine levels among the four groups were compared using the Kruskal–Wallis test, and pairwise comparisons were used to examine the differences between each pair of groups. Statistical significance was set at *p* < 0.05 for the two‐tailed analysis. Multiple test was constructed with a Bonferroni adjustment (*p* < 0.0083).

## 3. Results

Baseline characteristics of the 81 eyes in the four groups are listed in Table 1.

**Table 1:** Baseline characteristics of patients in the groups.

	PCV w/MH (*n* = 20)	PCV w/o MH (*n* = 19)	nAMD (*n* = 19)	Controls (*n* = 23)
Age (y) median (Q1, Q3)	65 (59, 77)	73 (67, 81)	77 (70, 87)	72 (69, 77)
p vs PCV w/MH		1.000	0.041	1.000
p vs PCV w/o MH			1.000	1.000
p vs nAMD				0.05

Gender (male: female)	14:6	10:9	16:3	13:10
Vision (Log MAR) mean ± SD	1.8 ± 0.8	0.7 ± 0.3	0.9 ± 0.5	0.6 ± 0.4
p vs PCV w/MH		< 0.001^∗^	0.003^∗^	< 0.001^∗^
p vs PCV w/o MH			1.000	1.000
p vs nAMD				0.747

Duration of vision reduced in months median (Q1, Q3)	2 (0.4, 12)	12 (1, 12)	12 (1, 12)	12 (12, 12)
p vs PCV w/MH		1.000	1.000	0.014
p vs PCV w/o MH			1.000	0.886
p vs nAMD				1.000

Abbreviation: MH, massive hemorrhage.

^∗^Statistical significance, *p* < 0.0083; gender showed no significance in the Kruskal–Wallis test.

The aqueous levels of the 10 cytokines (VEGF, IL‐6, IL‐8, MCP‐1, ICAM‐1, VCAM‐1, IP‐10, G‐CSF, IFN‐γ, IL‐10) of all groups are presented in Table 2 and are illustrated graphically in Figure 1. In order to magnify the difference in cytokine levels among PCV without massive hemorrhage, nAMD, and control, data of PCV with massive hemorrhage are not shown in Figure 2.

**Table 2:** The concentration of the 10 cytokines in the aqueous humor in the groups in pg/mL (median (Q1, Q3)).

	PCV w/MH (*n* = 20)	PCV w/o MH (*n* = 19)	nAMD (*n* = 19)	Controls (*n* = 23)
VEGF	5.20 (2.34, 16.51)	34.76 (12.67, 48.93)	43.88 (31.24, 65.58)	22.02 (13.74, 37.17)
p vs PCV w/MH		0.003^∗^	< 0.001^∗^	0.037
p vs PCV w/o MH			0.417	1.000
p vs nAMD				0.032

IL‐6	97.94 (39.66, 268.07)	6.80 (5.21, 10.41)	5.41 (3.18, 11.96)	7.40 (3.78, 21.02)
p vs PCV w/MH		< 0.001^∗^	< 0.001^∗^	< 0.001^∗^
p vs PCV w/o MH			1.000	1.000
p vs nAMD				1.000

IL‐8	386.44 (54.18, 1506.92)	7.22 (3.34, 12.10)	6.85 (4.16, 15.53)	4.15 (2.32, 5.68)
p vs PCV w/MH		< 0.001^∗^	< 0.001^∗^	< 0.001^∗^
p vs PCV w/o MH			1.000	0.313
p vs nAMD				0.403

MCP‐1	3865.28 (1612.22, 7381.82)	506.70 (337.96, 715.39)	465.29 (383.87, 600.83)	465.65 (339.37, 538.69)
p vs PCV w/MH		< 0.001^∗^	< 0.001^∗^	< 0.001^∗^
p vs PCV w/o MH			1.000	1.000
p vs nAMD				1.000

ICAM‐1	766.54 (297.01, 1193.39)	118.93 (34.23, 320.43)	75.28 (42.47, 136.70)	64.62 (25.49, 236.29)
p vs PCV w/MH		0.002^∗^	< 0.001^∗^	< 0.001^∗^
p vs PCV w/o MH			1.000	1.000
p vs nAMD				1.000

VCAM‐1	3413.66 (2111.35, 5152.33)	1466.95 (963.12, 2403.18)	1137.58 (869.84, 1870.97)	1049.52 (633.09, 1447.77)
p vs PCV w/MH		0.018	0.004^∗^	< 0.001^∗^
p vs PCV w/o MH			1.000	0.505
p vs nAMD				1.000

IP‐10	1251.84 (386.09, 3614.70)	77.49 (33.39, 149.23)	65.50 (26.37, 159.80)	52.33 (17.56, 88.86)
p vs PCV w/MH		< 0.001^∗^	< 0.001^∗^	< 0.001^∗^
p vs PCV w/o MH			1.000	1.000
p vs nAMD				1.000

G‐CSF	0.30 (0.00, 2.72)	0.46 (0.04, 0.61)	0.01 (0.00, 0.07)	0.00 (0.00, 0.02)
p vs PCV w/MH		1.000	0.153	0.034
p vs PCV w/o MH			0.032	0.005^∗^
p vs nAMD				1.000

IFN‐r	0.11 (0.01, 0.20)	0.18 (0.04, 0.86)	0.07 (0.02, 0.25)	0.00 (0.00, 0.16)
p vs PCV w/MH		1.000	1.000	0.257
p vs PCV w/o MH			0.944	0.007^∗^
p vs nAMD				0.415

IL‐10	0.74 (0.08, 1.71)	0.53 (0.16, 0.81)	0.07 (0.00, 0.53)	0.33 (0.00, 0.59)
p vs PCV w/MH		1.000	0.005^∗^	0.071
p vs PCV w/o MH			0.075	0.635
p vs nAMD				1.000

Abbreviation: MH, massive hemorrhage.

^∗^Statistical significance, *p* < 0.0083.

Figure 1Scatter plots with error bars represent median with an interquartile range. Aqueous levels of 10 cytokines in all groups.

**Figure:**
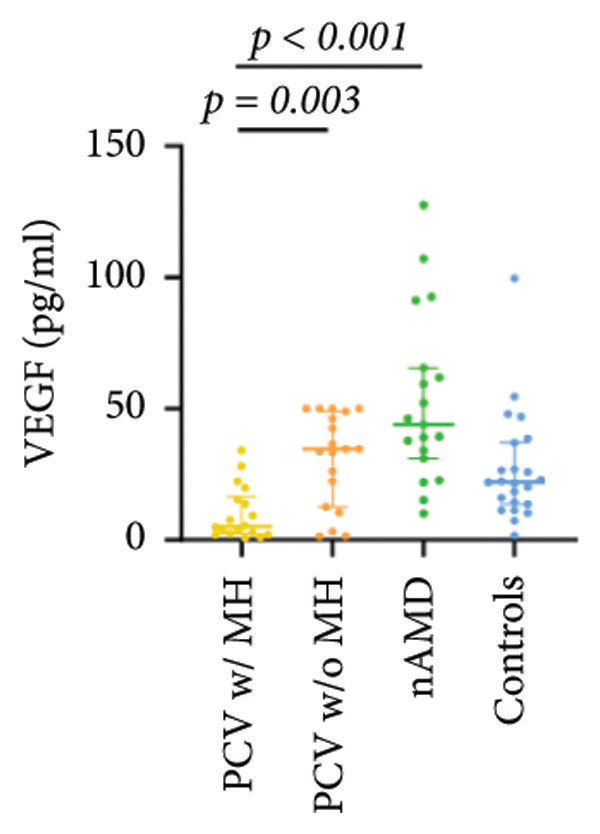
(a)

**Figure:**
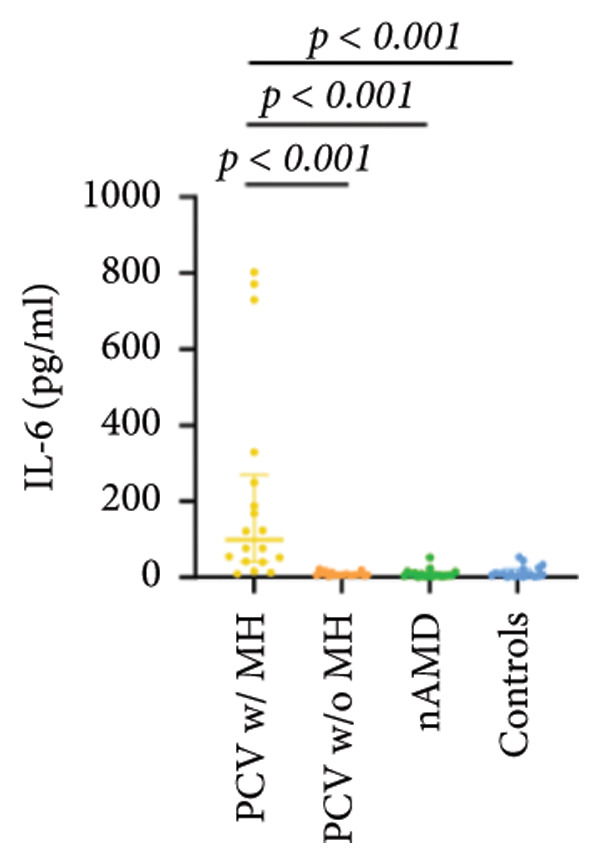
(b)

**Figure:**
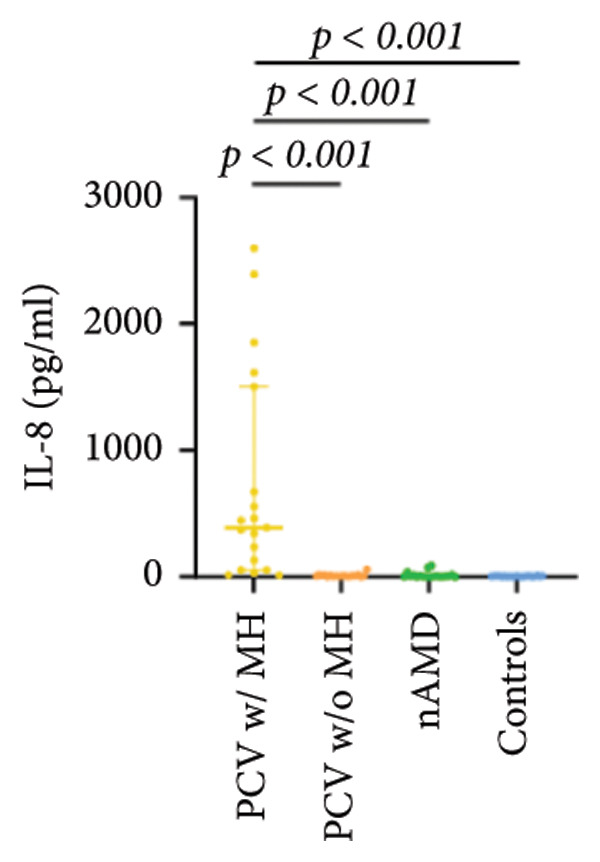
(c)

**Figure:**
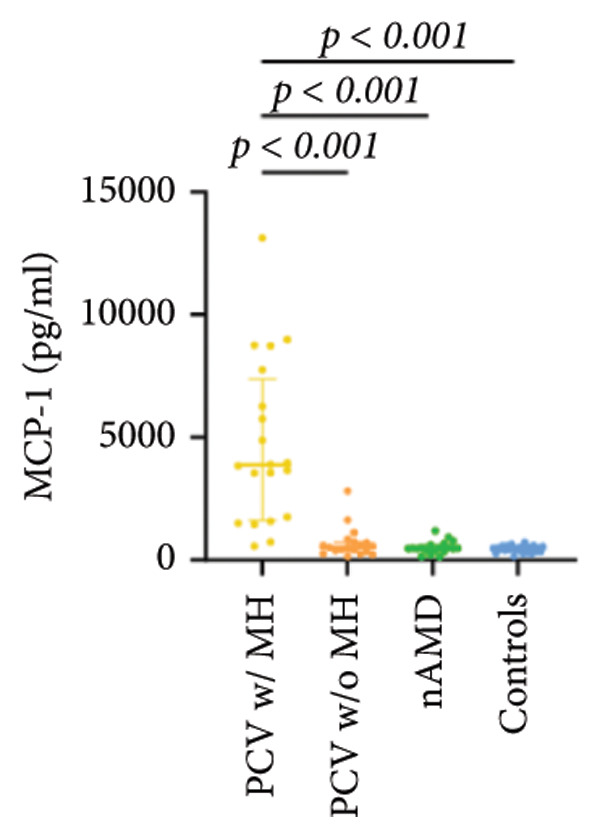
(d)

**Figure:**
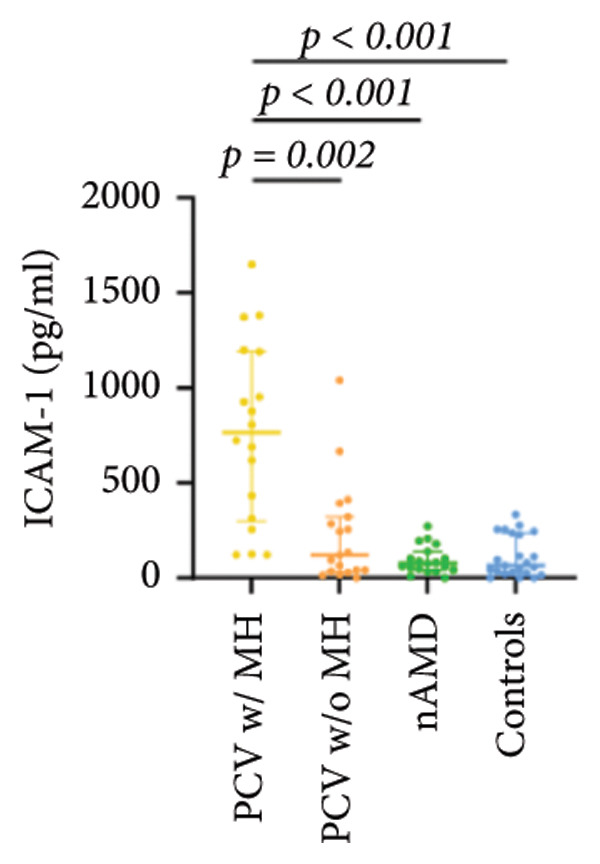
(e)

**Figure:**
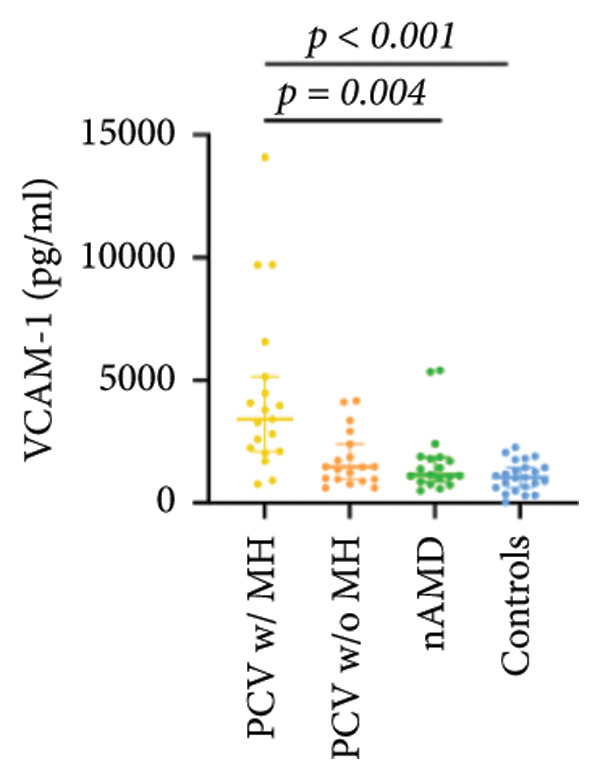
(f)

**Figure:**
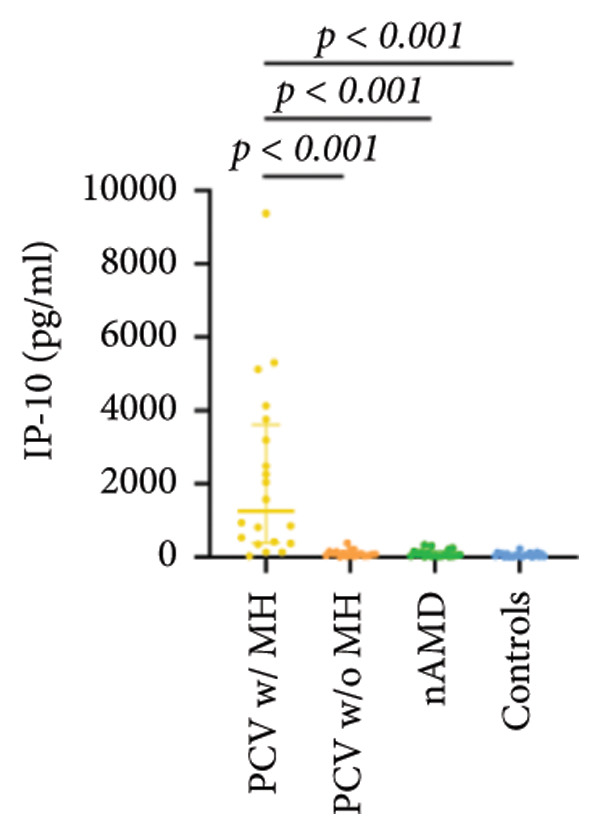
(g)

**Figure:**
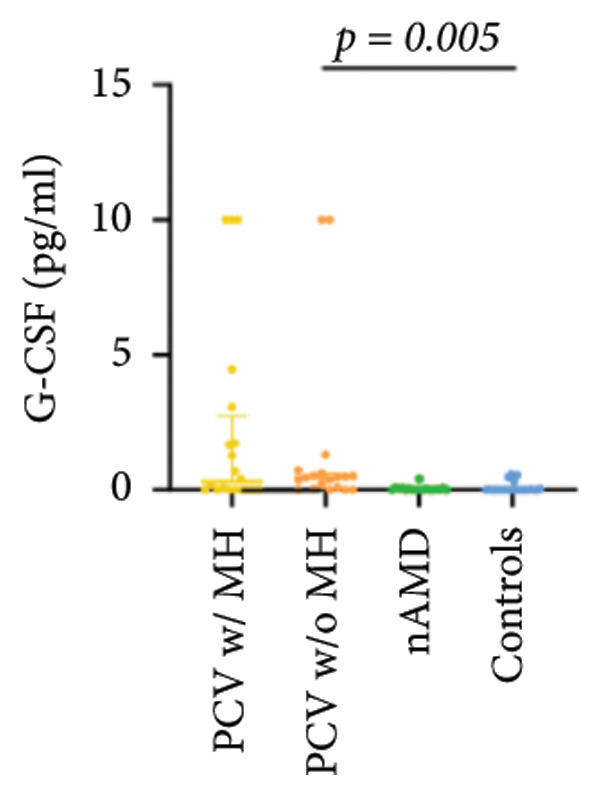
(h)

**Figure:**
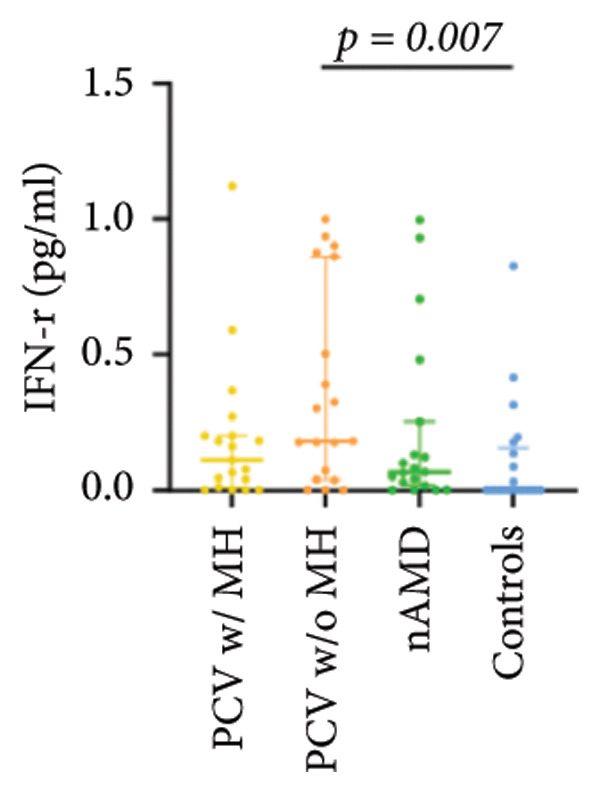
(i)

**Figure:**
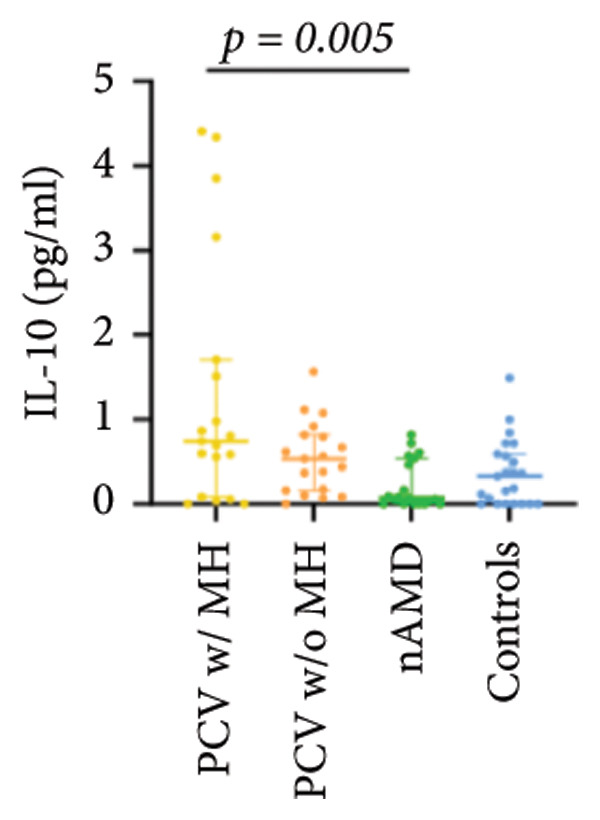
(j)

Figure 2Scatter plots with error bars represent median with an interquartile range. Aqueous levels of 10 cytokines in PCV without massive hemorrhage, nAMD, and control.

**Figure:**
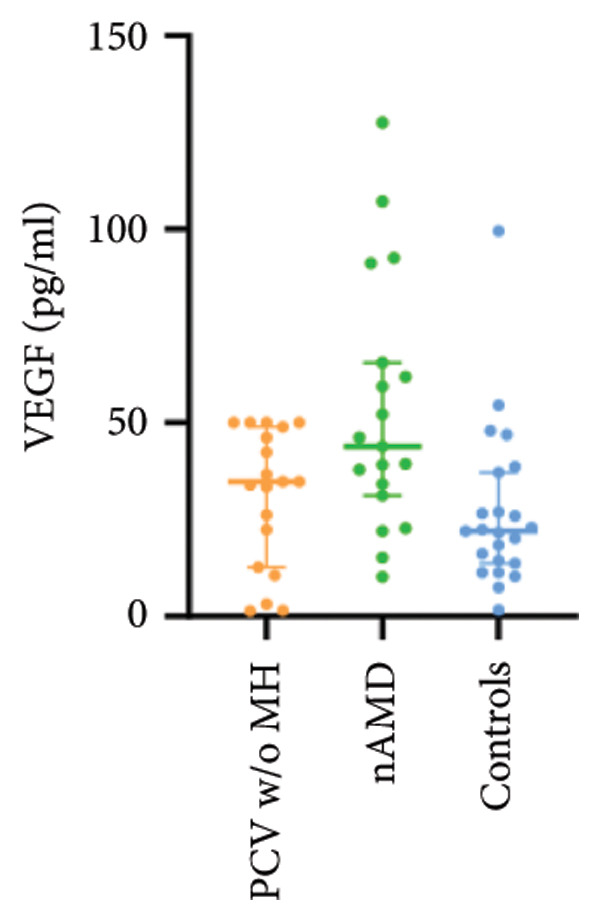
(a)

**Figure:**
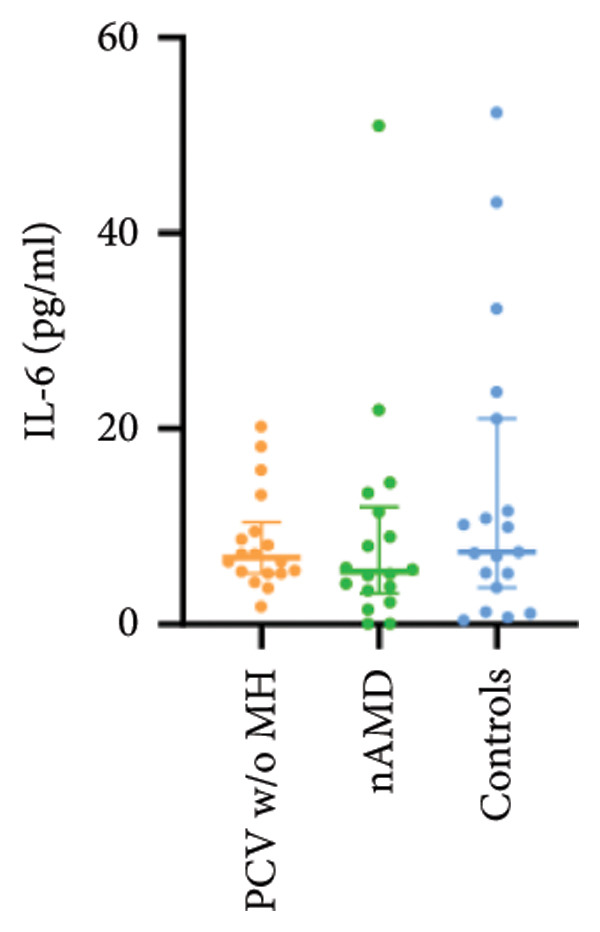
(b)

**Figure:**
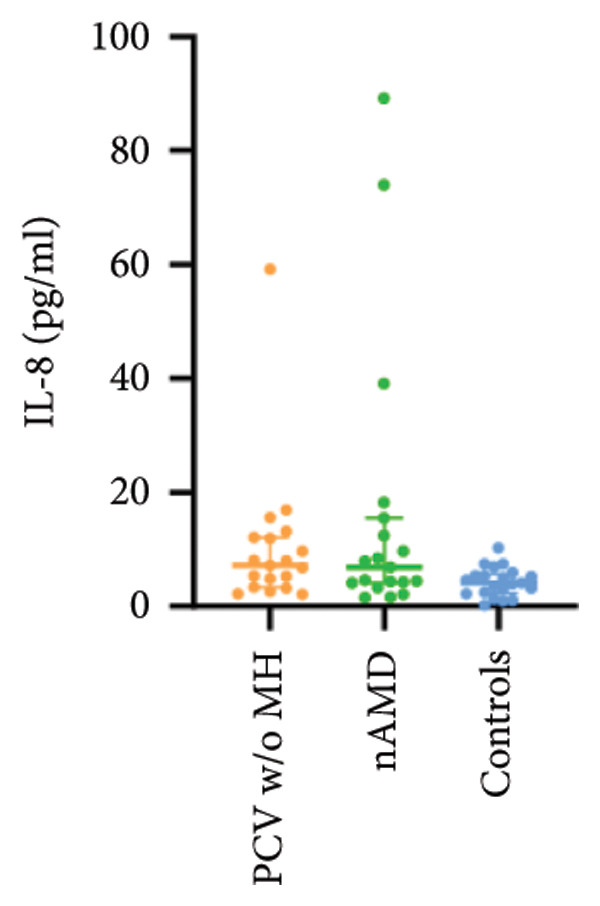
(c)

**Figure:**
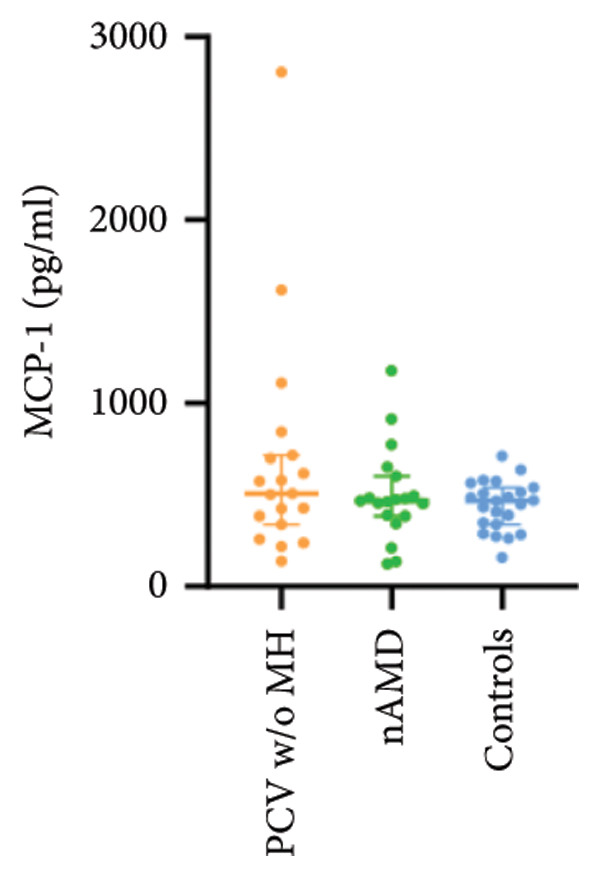
(d)

**Figure:**
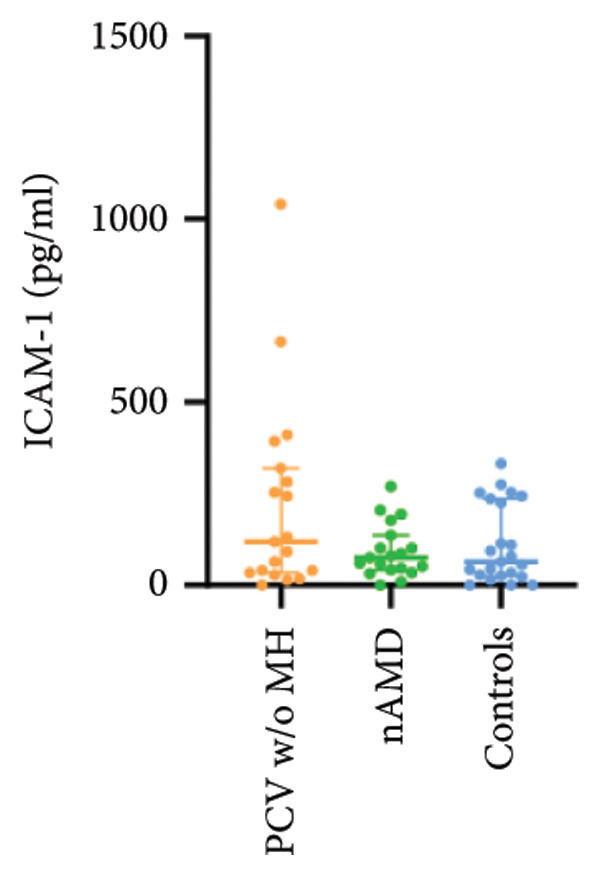
(e)

**Figure:**
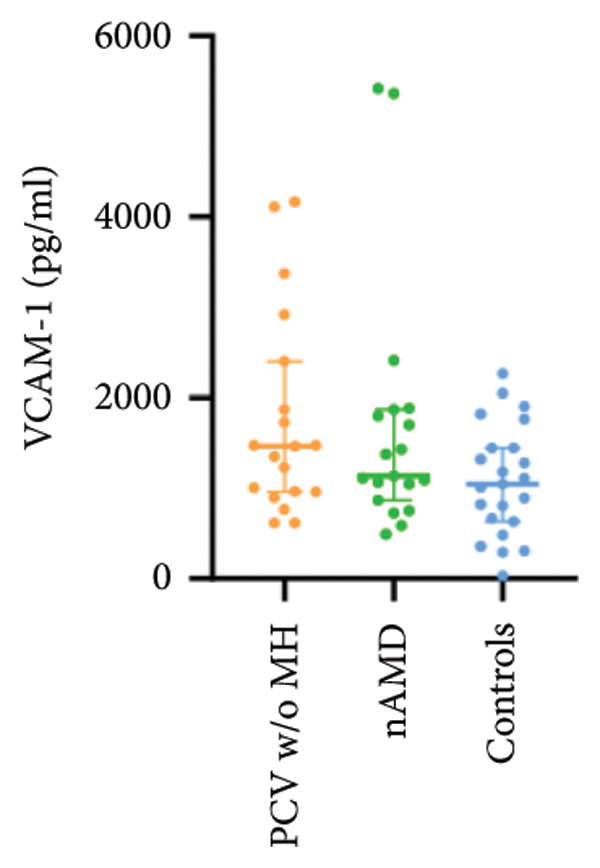
(f)

**Figure:**
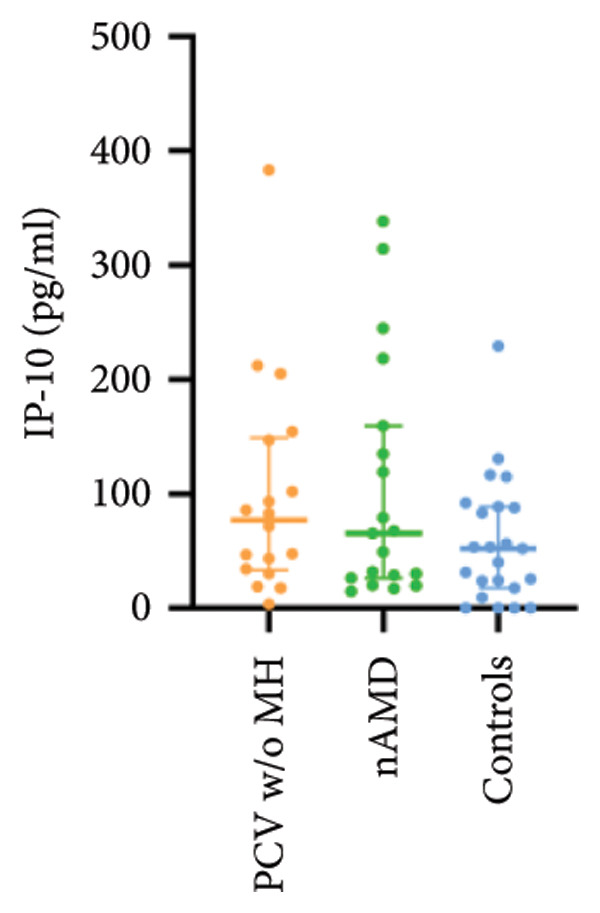
(g)

**Figure:**
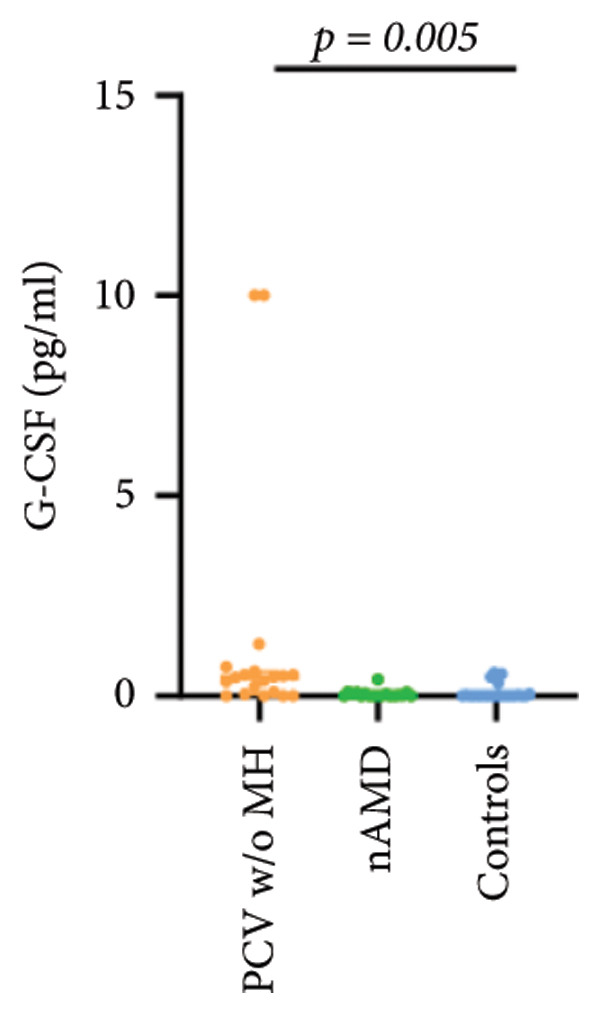
(h)

**Figure:**
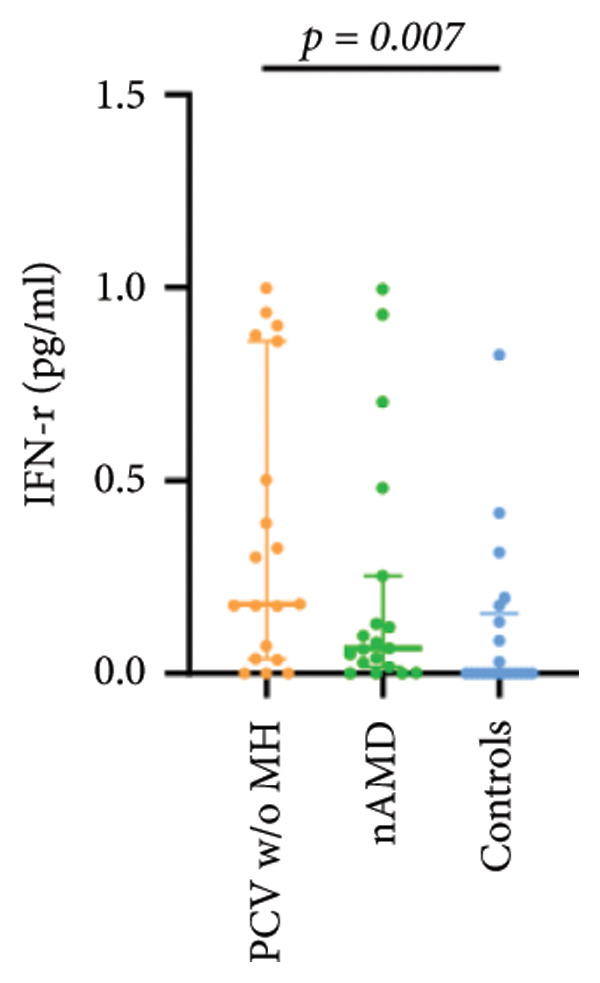
(i)

**Figure:**
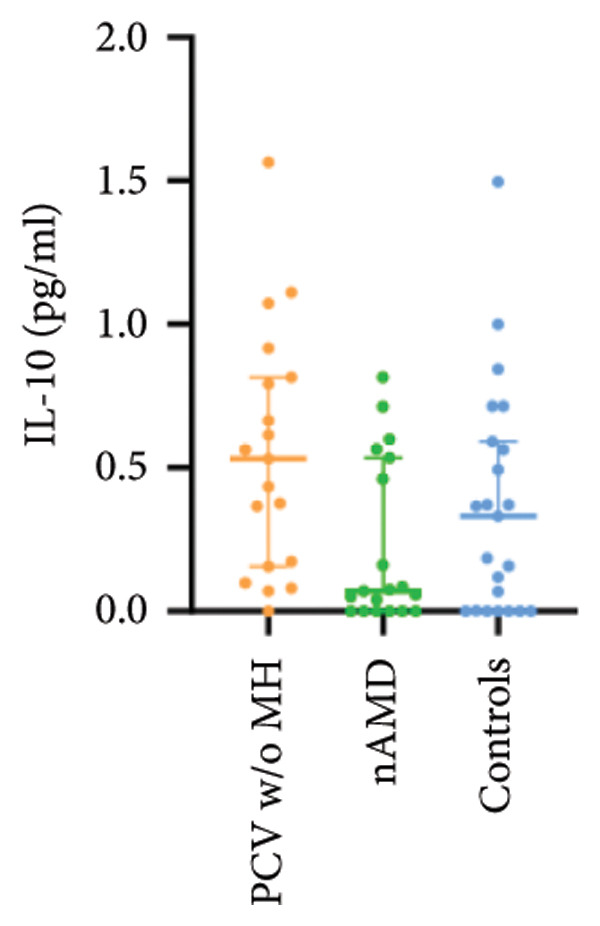
(j)

### 3.1. VEGF

VEGF levels in PCV with massive hemorrhage (median, 5.43 pg/mL) were significantly lower than that in PCV without massive hemorrhage (median, 34.76 pg/mL; *p* = 0.002) and typical nAMD (median, 43.88 pg/mL; *p* < 0.001). They were even lower than that in normal cataract controls (median, 22.02 pg/mL), although they did not reach the level of significance after Bonferroni adjustment (*p* = 0.003). VEGF levels in typical nAMD were higher than that in PCV without massive hemorrhage and normal cataract controls, but the difference did not reach the level of significance after Bonferroni adjustment.

### 3.2. IL‐6, IL‐8, MCP‐1, ICAM‐1, VCAM‐1, and IP‐10

IL‐6, IL‐8, MCP‐1, ICAM‐1, and IP‐10 levels were significantly higher in PCV with massive hemorrhage than that in the other three groups. VCAM‐1 levels were significantly higher in PCV with massive hemorrhage than that in typical nAMD and in control but did not reach the level of significance after Bonferroni adjustment between PCV with or without massive hemorrhage (*p* = 0.018).

### 3.3. G‐CSF, IFN‐γ, and IL‐10

G‐CSF and IFN‐γ levels were significantly higher in PCV without massive hemorrhage than that in control. IL‐10 levels were significantly higher in PCV with massive hemorrhage than that in typical nAMD. There was no statistical significance among the other groups.

## 4. Discussion

PCV has a variable clinical course. Some are more stable and favorable as compared with nAMD, while others have a devastating consequence resulting from massive hemorrhagic events [11, 12]. Uyama et al. [29] categorized the natural history of PCV into two distinct phenotypes: exudative type, characterized by the serous detachment of the RPE and neuroretina, or hemorrhagic type, characterized by hemorrhagic PED and subretinal hemorrhage. Hemorrhages may develop from the rupture of venules and occasionally arterioles. Furthermore, in an analysis subgrouping PCV into pachychoroid and nonpachychoroid types, patients in the pachychoroid PCV group were younger; all had hemorrhagic PED, with a higher prevalence of choroidal vascular hyperpermeability and hemorrhagic retinal detachment [30]. These findings suggest the heterogeneity of PCV. Whether PCV with or without massive hemorrhage are different phenotypes or within different stages of a continuous disease course is not clearly known. However, comparing the angiogenic and inflammatory cytokine profiles between PCV with and without massive hemorrhage and between PCV and typical AMD is of great significance. However, it must be noted that any study attempting to evaluate risk factors or biomarkers, which can help to differentiate PCV and nAMD, may face one core issue, that is, making a correct clinical diagnosis first. ICG has been the gold standard for the diagnosis of PCV; however, there is no universally accepted ICG definition of PCV [3, 6]. Overlap of imaging features between PCV and nAMD makes the diagnosis a difficult one. In one study, the concordance of PCV and nAMD diagnoses was 77% even among retinal specialists experienced in the management of PCV [31]. In 2021, the Asia‐Pacific Ocular Imaging Society PCV Workgroup developed and validated a set of diagnostic criteria not requiring ICGA for differentiating PCV from typical nAMD based on a combination of OCT and color fundus photography findings [4]. Therefore, in the present study, the differential diagnosis between PCV and nAMD was not merely based on ICG but rather based on multimodal imaging, which may add more accuracy to the differential diagnosis. However, it should be noted that if genetic analysis was performed, the classification may be different. Furthermore, in this study, we separated PCV with massive hemorrhage from PCV without massive hemorrhage because they are dramatically different phenotypes. Whereas, in the presence of a dense vitreous hemorrhage obscuring the media, ICG, OCT, or OCTA cannot be performed. PCV with massive hemorrhage was diagnosed on the basis of preoperative B ultrasonographic findings showing extensive subretinal hemorrhage, as well as intraoperative observation of both extensive subretinal hemorrhage and large hemorrhagic PED, with or without orange‐red subretinal elevations, which were all directly visualized after the neural retina was flipped in all cases with massive hemorrhage. However, it should also be noted that massive hemorrhage is not merely specific to PCV, and systemic factors such as the use of anticoagulants can significantly influence the likelihood of hemorrhage. Therefore, there was a likelihood of misclassification in these patients.

VEGF is the most well‐known factor in the pathogenesis of nAMD, and the advent of anti‐VEGF treatment has dramatically improved the prognosis of patients with nAMD. However, many studies have showed that patients with PCV may be refractory to anti‐VEGF monotherapy. Comparison of aqueous VEGF levels between PCV and nAMD patients was not in consistency in the previous literature. One study [23] reported significantly lower VEGF levels in eyes with PCV than those of nAMD, while the other three studies showed no significant difference in the aqueous levels of VEGF between PCV and nAMD patients [24–26]. Results of VEGF expression in surgically excised tissue specimens were also varied. In one study [22], the vascular endothelial cells of PCV were a lack of VEGF positivity, while in another report, a strong expression of VEGF positivity was found in the vascular endothelial cells of PCV‐associated membrane [32]. In the present study, the measurement result of VEGF in four different groups is of great interest. We found that VEGF levels in the aqueous humor were dramatically decreased in PCV with massive hemorrhage (median, 5.43 pg/mL) as compared with both PCV without massive hemorrhage (median, 34.76 pg/mL; *p* = 0.003) and typical nAMD (median, 43.88 pg/mL; *p* < 0.001) and even lower than those in the cataract controls (median, 22.02 pg/mL) although they did not reach the level of significance after the Bonferroni adjustment (*p* = 0.037). VEGF levels in typical nAMD were higher than that in PCV without massive hemorrhage and normal cataract controls, but the difference did not reach the level of significance after the Bonferroni adjustment. The variation of VEGF levels in the four groups reflects the extent of ischemia in the macular area. VEGF concentrations may peak just prior to neovascularization and decrease once the neovascular tissue becomes more mature and relieves ischemia. Nevertheless, VEGF levels in PCV with massive hemorrhage (median, 5.43 pg/mL) were even lower than those in the normal controls (median, 22.02 pg/mL; *p* = 0.037) and are somewhat unexpected. Whether there is a cause–effect relationship between the reduction of VEGF expression and massive hemorrhage is uncertain. On the one hand, decreased secretion of VEGF by local cells, RPE cells in particular, may precede massive hemorrhage. Since VEGF is an important trophic factor for choriocapillaris [33], lack of VEGF may cause dysfunction and disorganization of the choriocapillaris, which may subsequently result in hemodynamic abnormality in the choroid and, under some circumstances such as a sudden increased blood pressure, eventually lead to the rupture of choroidal vessels or polypoidal lesions causing massive hemorrhage. Interestingly, a decreased thickness of choriocapillaris has been found to be one of the key features of pachychoroid disease including PCV [34]. On the other hand, the reduction of VEGF expression could be secondary to massive hemorrhage. Since VEGF is not only an angiogenic factor but also a strong permeability factor, which induces the blood–retina barrier breakdown. It might be possible that the drop of VEGF is due to a protective feedback after massive hemorrhage, which can decrease the vascular permeability to alleviate bleeding. Another explanation is that the sudden sub‐RPE hemorrhage can lead to RPE malfunction, which might cut down the synthesis and secretion of VEGF. Furthermore, we found that there was no significant difference in VEGF levels between PCV without massive hemorrhage and control, but both were lower than in nAMD. These findings support the concept that PCV may be less VEGF‐driven than nAMD and could explain why PCV may be less responsive to anti‐VEGF monotherapy than nAMD.

Inflammation also plays an important role in both PCV and nAMD [6, 27]. There have been several studies comparing the differences in inflammatory cytokine levels in aqueous humor between eyes with PCV and in those with nAMD, but in all of these studies, none of the measured cytokines showed significant difference between PCV and nAMD [24–27]. These results added more elusiveness to the unsettled question regarding whether PCV is a distinct entity or a variant of nAMD [5, 6]. In the present study, we found that multiple inflammatory cytokines were significantly elevated in PCV with massive hemorrhage as compared with the other three groups but of no significant differences between the other three groups (IL‐6, IL‐8, MCP‐1, ICAM‐1, IP‐10). However, whether elevation of these inflammatory cytokines precedes or are secondary to massive hemorrhage in PCV is not fully understood. In eyes with massive hemorrhage, there is presumably a profound breakdown of the blood–ocular barrier and blood components may have leaked into the anterior chamber. A recent study showed that aqueous humor protein concentration, pseudophakia, and pupil dilation with phenylephrine/tropicamide are important confounding factors for aqueous humor protein analyses. When these factors are considered, aqueous humor analyses can more clearly reveal disease‐relevant factors [35]. Unfortunately, these confounding factors were not considered in our study. The dramatic elevation of multiple cytokines (IL‐6, IL‐8, MCP‐1, etc.) in the PCV w/MH group may simply reflect a nonspecific influx of proteins due to barrier breakdown. However, it should be noted that not all 10 measured cytokines were consistently elevated, and previous studies have shown that inflammation may be actively involved in the pathogenesis of massive hemorrhage in PCV. In a clinicopathologic study of a patient with multiple recurrent serosanguineous PED syndrome, which is a former name of PCV, intense infiltration of plasma cells and lymphocytes was found in the choroid and fibrovascular tissue in the subretinal space and within Bruch’s membrane [28]. The author speculated that these inflammatory cells were unlikely secondary to the hemorrhage itself, as extensive inflammatory infiltrates have not been observed in patients with massive hemorrhage from other causes such as diabetic retinopathy. In a study comparing the phenotype of a PCV animal model [36], the mouse with severe PCV phenotype exhibited prominent immune complex deposition, complement activation, and infiltration of inflammatory cells, compared to those with weak PCV phenotype. The authors proposed that the progression stage of PCV should be driven by inflammatory cascades. These results together with our findings suggest that inflammation should be given attention in the future research regarding the pathogenesis of advancement of PCV. In addition, it was also interesting to look at if there was a significant difference in the aqueous level of any inflammatory cytokines between PCV without massive hemorrhage and typical nAMD. We found that G‐CSF and IL‐10 were 10‐folds higher in PCV without massive hemorrhage than in typical nAMD, although the absolute values of these two cytokines in both groups were low (< 1 pg/mL) and the differences did not reach the level of significance after the Bonferroni adjustment. Interestingly, aqueous levels of G‐CSF and IL‐10 were similar in eyes of PCV with or without massive hemorrhage, indicating that levels of these two cytokines are relatively stable in different phenotypes or different stages of PCV, but higher than typical nAMD. Whether these two cytokines (G‐CSF, IL‐10) can be used to differentiate PCV from typical nAMD still need further validation.

There are several limitations of the present study that should be mentioned. Firstly, this is a retrospective study with relatively small sample sizes, which limits the statistical power and the robustness of the conclusions. Secondly, we did not measure the major confounding factors identified in the literature, such as the total aqueous humor protein concentration and lens status. The subretinal hemorrhage and the potentially larger lesion size in PCV with massive hemorrhage may influence the measurement results. These limit the interpretation of the findings, especially concerning the inflammatory cytokines. Thus, this study should be considered as a pilot study whose conclusions require further validation in the future. Thirdly, although the differential diagnosis between PCV and nAMD was based on multimodal imaging in this study, we could not exclude the possibility of misclassification because there were overlapping features in some cases. In particular, in PCV patients with a dense vitreous hemorrhage obscuring the media, ICG and OCT cannot be undertaken and the diagnosis of PCV with massive hemorrhage was made on the basis of preoperative B ultrasonography as well as intraoperative findings of both extensive subretinal hemorrhage and large hemorrhagic PED, with or without orange‐red subretinal elevations. Whereas massive hemorrhage is not as specific to PCV as often assumed and systemic factors such as the use of anticoagulants significantly influence the likelihood of hemorrhage. Furthermore, classification based on the associated genetic background rather than merely clinical presentations may have more accuracy but not performed in this study. All of these may introduce a degree of diagnostic uncertainty that could affect group homogeneity. Lastly, intraocular cytokine levels were measured with samples from aqueous humor instead of vitreous humor. In patients with a small lesion size, cytokine levels in the aqueous humor may not be sensitively elevated to reflect the true difference among groups. Measurements below the lowest concentration on the standard curve for each cytokine were extrapolated values and might be less reliable. Due to the limited volume of the aqueous humor samples, we only analyzed 10 cytokines, while there are many other cytokines that may actively involve in the disease but not included in our study.

Nevertheless, in this study, we specifically characterize the aqueous cytokine profile in the hemorrhagic phenotype of PCV and found a dramatic decrease of VEGF and significant elevation of several inflammatory cytokines in PCV with massive hemorrhage compared to PCV without massive hemorrhage, typical nAMD, and control eyes. Our findings may provide a clue to understanding the pathogenesis of massive hemorrhage in PCV that this catastrophic event seems to be less likely VEGF‐driven and inflammation may actively involve in this process.

## Conflicts of Interest

The authors declare no conflicts of interest.

## Funding

This study was supported by Peking University‐Golden Resource “Tengyun Clinical Research Program,” Institute of Advanced Clinical Medicine, Peking University (No. TY2025005).

## Data Availability

The data that support the findings of this study are available from the corresponding author upon reasonable request.
